# Conflict-related trauma and bereavement: exploring differential symptom profiles of prolonged grief and posttraumatic stress disorder

**DOI:** 10.1186/s12888-017-1286-2

**Published:** 2017-03-29

**Authors:** Carina Heeke, Nadine Stammel, Manuel Heinrich, Christine Knaevelsrud

**Affiliations:** 1 Department of Clinical-Psychological Intervention, Freie Universität Berlin, Habelschwerdter Allee 45, 14195 Berlin, Germany; 2 Center Ueberleben gGmbH, Turmstr. 21, 10559 Berlin, Germany

**Keywords:** Prolonged grief, Posttraumatic stress disorder, Trauma, Bereavement, Armed conflict

## Abstract

**Background:**

Exposure to trauma and bereavement is common in conflict-affected regions. Previous research suggests considerable heterogeneity in responses to trauma and loss with varying symptom representations. The purpose of the current study was to (1) identify classes of prolonged grief disorder (PGD) and posttraumatic stress disorder (PTSD) symptom profiles among individuals who were exposed to both trauma and loss due to the Colombian armed conflict and (2) to examine whether sociodemographic, loss and trauma-related characteristics could predict class membership.

**Methods:**

Three hundred eight victims of internal displacement who had experienced trauma and loss were assessed through measures of PGD (PG-13), PTSD (PCL-C), and social support (DUKE-UNC). Latent class analysis (LCA) was performed to analyze differential profiles by symptoms of PGD and PTSD and multinomial logistic regression was used to analyze predictors of class membership.

**Results:**

LCA revealed a four-class solution: a resilient class (23.6%), a PTSD-class (23.3%), a predominately PGD class (25.3%) and a high distress-class with overall high values of PGD and PTSD (27.8%). Relative to the resilient class, membership to the PGD class was predicted by the loss of a close family member and the exposure to a higher number of assaultive traumatic events, whereas membership to the PTSD class was predicted by the perception of less social support. Compared to the resilient class, participants in the high distress-class were more likely to be female, to have lost a close relative, experienced more accidental and assaultive traumatic events, and perceived less social support.

**Discussion:**

Specific symptom profiles emerged following exposure to trauma and loss within the context of the Colombian armed conflict. Profiles were associated with distinct types of traumatic experiences, the degree of closeness to the person lost, the amount of social support perceived, and gender. The results have implications for identifying distressed subgroups and informing interventions in accordance with the patient’s symptom profile.

## Background

Worldwide, people are exposed to trauma and bereavement in the context of violent conflicts and systematic human rights violations. The high rates of posttraumatic stress disorder (PTSD) and depression as a consequence of traumatic experiences within these conflicts have been documented in several studies [1].

A large proportion of human war casualties are civilians. Traumatic experiences within violent conflicts therefore often involve the loss of significant others and studies have addressed maladaptive grief in conflict-affected populations only recently. Evidence suggests that a significant number of individuals experience persistent distressing grief symptoms after the loss of a loved one. Prolonged grief disorder (PGD) as a maladaptive reaction to loss is marked by separation distress, feelings of emptiness, and difficulties in moving on over a period of at least 6 months [2]. While PGD was rejected as a diagnosis in the Diagnostic and Statistical Manual of Mental Disorders (DSM-5, [3]) and has only been introduced as a condition for further study (“persistent bereavement related disorder”), PGD is proposed for inclusion in the forthcoming edition of the International Classification of Diseases (ICD-11; [4]).

It is estimated that 2.4–10% of bereaved individuals develop PGD after the death of a significant other [5–7]. Evidence suggests that losing someone from a violent cause, i.e. by homicide, suicide or accident, is associated with an elevated risk for both PGD and PTSD [8, 9]. Both disorders are aetiologically defined by a potentially traumatizing experience and are assumed to occur due to insufficient integration of the experience into the autobiographical knowledge base [10, 11]. At the symptom level, both disorders differ with respect to some central elements. While PTSD is characterized by intrusions of the event, avoidance of reminders of the event, and persistent symptoms of increased arousal according to DSM-IV, the dominant element in PGD is separation distress in relation to the lost person [2, 12]. Furthermore, whereas in PTSD negative appraisals refer to the potential reoccurrence of danger, in PGD, negative appraisals are concerned with the impact of the loss on the self and the future [10, 11]. However, in how far PTSD and PGD are distinguishable in cases of traumatic losses, is still a matter of debate. PGD rates vary between 8 and 38% between different violent conflict contexts [13–17]. Additional traumatic stressors occurring during conflict may exacerbate the grieving process [15].

Research indicates considerable heterogeneity in responses to trauma and to bereavement with varying symptom representations. Galatzer-Levy and Bryant [18] stated that within the new DSM-5 symptom criteria, 636.120 combinations of PTSD symptom profiles are possible. The identification of subgroups characterized by specific profiles may broaden the knowledge on clinical manifestations of responses to trauma and bereavement and may have implications for forming interventions due to potential patterns of comorbidity.

To our knowledge, only two studies evaluated symptom profiles of psychopathology among individuals confronted with a violent loss using latent class analysis. Nickerson et al. [19] determined subgroups of PGD and PTSD profiles among 248 Mandaean refugees exposed to significant trauma and loss. A four-class solution emerged with a combined PGD/PTSD class, a predominately PTSD class, a predominately PGD class and a resilient class. A relevant factor in predicting membership to the combined PGD/PTSD-class was the exposure to traumatic loss, whereas problems related to adaption difficulties since relocation predicted membership to the PGD-class. Boelen et al. [20] conducted a similar study identifying subgroups of PGD and depression symptom profiles among 245 individuals confronted with an unnatural or violent death. LCA revealed a three-class solution with a resilient class, a predominately PGD class and a combined PGD/depression class. PGD-class membership was mainly predicted by negative cognitions about the self and life. Thus, although partly focusing on different disorders (PTSD vs. depression), both studies found similar symptom profiles that were not only associated with the overall severity of distress but also with the dominance of a particular syndrome in response to the loss, i.e. some with dominant PGD and others with both elevated PGD and PTSD/ depression [19, 20].

To replicate and extend previous findings by Nickerson et al. [19], the current study seeks to identify differential symptom profiles of PGD and PTSD among individuals who were exposed to both trauma and loss. Similar to these previous results, we expected a four class-solution with classes separable both by symptom severity and disorder-specific response. Participants for this study were Colombian survivors of internal displacement. Colombia has been faced with an armed conflict for 60 years. More than six million people have been forcibly displaced while thousands have been tortured, kidnapped and forcibly recruited to join the armed forces [21]. More than 220.000 people have lost their lives in the course of the armed conflict, leaving behind considerable numbers of bereaved individuals [22].

Our second goal was to identify variables that are associated with group membership by focusing on socio-demographic and loss-related characteristics as predictors. Prior research showed that the loss of a close family member was associated with higher PGD symptom severity than the loss of a distant family member [13, 23]. For PTSD, a dose-response-relationship between the number of traumatic events and PTSD is assumed [24]. However, assaultive traumatic events such as combat experiences or physical attacks are expected to be more harmful than other distressing events such as accidents or natural disasters [25]. The lack of social support has been identified as one of the major risk factors of PGD and PTSD [26, 27]. Its association with specific symptom profiles was assessed in the current study. Finally, forced disappearance is a common phenomenon within armed conflicts, in particular in Colombia. There is, however, inconclusive evidence whether relatives of individuals who have disappeared experience more severe mental health consequences than bereaved individuals do (e.g. [28, 29, 30]). Whether the type of loss (deceased vs. disappeared) has an impact on class membership will be explored further on.

In accordance with these previous findings, we hypothesized that classes would differ by trauma exposure and the closeness to the person lost. Individuals exposed to a higher number of assaultive traumatic events would be more likely to exhibit PTSD whereas individuals who had lost a close relative would be more likely to have PGD. Furthermore, we expected that individuals perceiving a high amount of social support would exhibit low PGD and PTSD symptom severity.

## Methods

The current study is part of a larger cross-sectional study on effects of internal displacement on the mental health and readiness to reconciliation among conflict-affected Colombians [31]. Data assessment for the current study was conducted in 2012. The study was approved by the ethics commission of the Freie Universität Berlin. Structured face-to-face interviews were conducted by three experienced Colombian clinical psychologists (M.A.) who had received a two-week training on the use of questionnaire measures and interview techniques and were supervised on a regular basis by the study coordinators (CH).

### Participants

Potential participants were randomly selected from a list of 1898 persons affiliated with our partner organization *Tierra y Vida*, a local NGO assisting displaced persons in the process of claiming the restitution of their land. Inclusion criteria required potential participants to be at least 18 years of age and to have experienced internal displacement within the armed conflict. No further exclusion criteria were applied. Of the initially 952 randomly selected persons, 498 persons could not be contacted due to outdated contact information or rejected participation. Participants were provided with a full description of the study and informed consent was obtained. Altogether, 454 persons were interviewed. In accordance with the purposes of this study, only participants who had experienced the loss of a significant other due to the armed conflict were included in the analysis (*N* = 308).

### Instruments

#### PGD

Symptoms of prolonged grief were measured using the Prolonged Grief −13 (PG-13), a screening instrument for PGD [32]. The PG-13 consists of two separation distress symptoms (“longing and yearning” and “sorrow or pangs of grief”) and nine “cognitive, emotional and behavioral” items. Prigerson et al. [2] provided psychometric validation. As no validated Spanish version of the PG-13 exists, it was translated into Spanish by a Colombian psychologist and back-translated by a psychologist who was unfamiliar with the original version. Discrepancies were discussed within a group of local psychologists and adaptions were made when necessary. Latent class analysis (LCA) uses binary indicators to identify patterns of responses. To this end, items that were originally scaled on a 5 point Likert scale, were dichotomized and a symptom was considered *absent* when occurring “not at all”, “at least once/slightly” or “at least once a week/somewhat” and *present* when occurring “at least once a day/quite a bit” or “several times a day/overwhelmingly” as suggested by the authors of the questionnaire [32]. Additionally, a set of loss-specific questions regarding the relationship to the lost person and the time elapsed since the loss were administered.

#### PTSD and traumatic events

PTSD was assessed using the PTSD Checklist-Civilian version (PCL-C), a 17-item self-report questionnaire based on the DSM-IV [33]. The PCL-C has been proven to be a valid and reliable screening instrument through a number of studies [34, 35]. The Spanish version of the instrument was validated for use among Hispanics [36] and was widely used in different Latin American populations (e.g. [37]). Originally scaled on a 5 point Likert scale, items were dichotomized and considered as *absent* when occurring *not at all* or *a little bit* and *present* when occurring *moderately*, *quite a bit* or *extremely* in accordance with the authors’ guidelines [33].

Traumatic events were assessed using an adjusted list based on two standardized instruments, the Harvard Trauma Questionnaire [38] and the Posttraumatic Diagnostic Scale [39], altogether assessing 23 traumatic events as reported by Nickerson et al. [40] and including one item allowing participants to indicate an additional traumatic event. Participants were asked whether they had personally experienced or witnessed the event. Traumatic events were divided into two groups: One group comprised traumatic events that included interpersonal, intentional violence, referred to as “assaultive violence”. The second comprised other traumatic events such as “natural disaster” or “serious accident” and was referred to as “other injury or shocking events”. This classification was based on a previous approach by Breslau and colleagues [25].

#### Social support

Perceived social support was measured using the validated Spanish translation of the DUKE-UNC Functional Social Support Questionnaire (DUKE-UNC-11; [41, 42]). The instrument comprises 11 items assessed on a 5-point Likert scale ranging from 1 (*much less than I would like*) to 5 (*as much as I would like*).

### Statistical analyses

LCA was conducted to identify different subgroups of individuals marked by comparable patterns of PTSD and PGD symptoms. Optimal number of classes was determined by taking several statistical criteria into account [43]. For each k class solution, Aikaike Information Criterion (AIC), Bayesian Information Criterion (BIC) and adjusted Bayesian Information Criterion (aBIC) were evaluated with lower values indicating better fit. In addition, bootstrap likelihood ratio test (BLRT) as well as the Vuong-Lo-Mendell-Rubin LRT were performed. A significant LRT for a *k* class solution suggests that the *k* class model describes the data better than the *k-1* class solution [44]. Entropy reflects the indication of classification accuracy of the model with values close to 1 indicating higher accuracy in class assignment. Consideration was moreover given to the size and interpretability of the distinct classes [45]. To compare PTSD and prolonged grief symptom severity between classes, variables were treated as outcome measure with unequal means and variances, and were compared using the three-step approach while inaccuracy of class assignment was taken into account [46]. Multinomial logistic regression was performed to assess predictors of class membership using the 3-step approach as suggested by Vermunt [47]. This approach takes inaccuracy of class assignment into account when assessing latent class predictors in multi-nominal regressions [47, 48]. Predictors included sociodemographic characteristics (gender, years of education), the number of assaultive and accidental traumatic events, the relationship to the person lost (close/ first grade vs. distant family member/friend), how the loss happened (deceased vs. disappeared), the time elapsed since the loss (in years) as well as the amount of perceived social support. Analyses were performed using *M*Plus 7 [45].

Missing values on latent class indicators and distal outcomes were dealt with using full information maximum likelihood estimation as implemented in *M*Plus. Missing values on predictors of class-membership were dealt with using EM-based single value imputation as implemented in SPSS to avoid listwise deletion.

## Results

### Sociodemographic characteristics

The sample consisted of *N* = 308 (190 females, 61.7%) internally displaced Colombians who had been exposed to both trauma and loss in the context of the armed conflict. Age ranged from 19 to 85 years (*M* = 48.5, *SD* = 12.7). Three quarters of the sample had lost a relative or friend to conflict-related violence. The remaining cases had lost a relative or friend to disappearance and did therefore not know whether their loved one was still alive. Participants in the study were highly exposed to traumatic events with an average of six “assaultive” (*SD* = 2.7, range: 1–13) and an average of four (*SD* = 1.9, range: 0–8) “accidental or other shocking events” (see Table 1).

**Table 1:** Sociodemographic characteristics

Characteristics		
Gender		
Female	*n* (%)	190 (61.7)
Age	*M* (*SD*)	48.5 (12.7)
Education (years)	*M* (*SD*)	5.5 (3.8)
Relationship to the loss^c^		
Close relative^a^	*n* (%)	214 (69.5)
Distant relative or friend^b^	*n* (%)	94 (30.5)
Time since loss (years)^c^	*M* (*SD*)	12.4 (7.2)
Type of loss^c^		
Deceased	*n* (%)	231 (75.0)
Disappeared	*n* (%)	77 (25.0)
Traumatic events		
Assaultive	*M* (*SD*)	6.1 (2.7)
Accidental	*M* (*SD*)	4.3 (1.9)

^a^partner, child, parent, sibling; ^b^aunt/uncle, grandparents, good friend; ^c^Number after EM imputation

### Latent class analysis

LCA started by fitting a one class solution and successively an increasing number of classes. We stopped with estimating a six-class solution since number of boundaries increased and visual inspection indicated that interpretability and separability of classes was low. In addition, the best loglikelihood value was not replicated. The VLMR-LRT was non-significant when fitting the three-class solution and thus favored the two-class model. However, since AIC, BIC and adjusted BIC decreased successively to higher number of classes, the two-class solution was dismissed. Fit indices did not support a three-class solution compared to other models. Although the five-class solution had lower AIC, adjusted BIC and higher entropy than the three- and four-class solution, it also exhibited a significant amount of boundaries indicating the extraction of too many classes [44]. The BIC showed the lowest value in the four-class solution. The four-class solution showed good interpretability in addition to the absence of boundaries, as well as a good entropy (0.86) and was therefore preferred. Fit indices for the 1 to 5 class solutions are presented in Table 2.

**Table 2:** Goodness of fit statistics for 1 to 5 class solutions

Number of classes	AIC	BIC	a-BIC	VLMRLRT	BLRT	Entropy
1	11,123.51	11,227.95	11,139.15	na	na	na
2	10,123.68	10,336.29	10,155.51	< .001	< .001	.877
3	9842.82	10,163.61	9890.86	ns	< .001	.848
4	*9693.86*	*10,122.82*	*9758.09*	*ns*	*< .001*	*.860*
5	9617.64	10,154.78	9698.07	ns	< .001	.901

*AIC* Aikaike Information Criterion, *BIC* Bayesian Information Criterion; *aBIC* adjusted BIC, *VLMLRT* Vuong-Lo-Mendell-Rubin likelihood ratio test, *BLRT* bootstrap likelihood ratio test

When interpreting LCA profiles, probabilities of symptom presentation ≥.70 were considered high and ≤.30 as low [49]. Classes were then considered homogenous regarding this symptom.

**Fig. 1:**
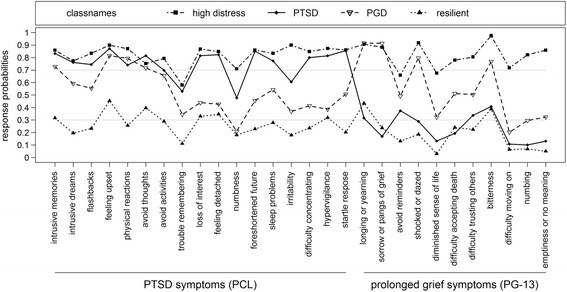
Estimated symptom probabilities for the four- class solution

The four-class solution comprised a predominately PTSD-class (23.3%), a predominately PGD class (25.3%), a high distress class with overall high item probabilities of PGD and PTSD (27.8%) and a resilient class (23.6%). Class 1 (PTSD: 23.3%) had high probabilities for almost all PTSD symptoms with the exception of “trouble remembering”, “numbness” and “irritability”. Moderate to low probabilities were evidenced for all PGD symptoms. In class 2 (PGD: 25.3%) high probabilities for “longing and yearning”, “sorrow or pangs of grief” as well as “shocked or dazed” and “bitterness” in relation to the loss were observed. High probabilities in this class were also found for the PTSD symptoms “intrusive memories”, “feeling upset”, “physical reactions” and “avoid thoughts”. Class 3 (high distress: 27.8%) showed high probabilities for all PTSD symptoms with the exception of “trouble remembering” and almost all PGD symptoms with the exception of “avoidance of reminders” and “diminished sense of life”. Class 4 (resilient: 23.6%) was characterized by moderate to low probabilities for all symptoms. Item response probabilities for symptomatic ratings are displayed for each class separately in Fig. 1.

### PGD and PTSD symptom severity in classes

The overall test of equality of means using the three-step approach indicated significant differences between the classes for PTSD, *χ* ^2^(df = 3) = 525.95, *p* < .001 and prolonged grief, *χ* ^2^(df = 3) = 562.39, *p* < .001. For PTSD symptom severity, the high distress class showed the highest mean (*M* = 61.55), followed by the PTSD class (*M* = 56.62), the PGD class (*M* = 45.49) and the resilient class (*M* = 32.47). For prolonged grief, the high distress class showed the highest mean (*M* = 45.41), followed by the grief class (*M* = 37.23), the PTSD class (*M* = 27.33) and the resilient class (*M* = 22.53). All pairwise comparisons for both outcomes were significant with *p* ≤ .001.

### Multinomial logistic regression

Predictors of class membership are displayed in Table 3. Compared to the resilient class, the likelihood for membership in the high distress class was higher for females, for those exposed to higher amount of types of assaultive and accidental traumatic events, by the loss of a close family member and less social support. Relative to the resilient class, membership to the PTSD class was predicted by less perceived social support. In contrast, membership to the PGD class was predicted by the number of assaultive traumatic events and members were more likely to have lost a close relative. Finally, comparing membership to the PGD class with the PTSD class, membership to the PGD class was predicted by the loss of a close family member and by less time that had elapsed since the loss.

**Table 3:** Multinomial logistic regression predicting class membership

	Estimates	SE	OR	95%-CI	Two-tailed *p*
High distress vs. Resilient (reference)					
Male gender^a^	−0.94	0.44	*0.39*	0.17–0.92	.032
Assaultive TE	0.21	0.09	*1.23*	1.04 –1.45	.017
Accidental TE	0.37	0.12	*1.44*	1.14 – 1.83	.003
Time elapsed since loss (years)	−0.06	0.03	0.95	0.89–1.01	.078
Distant family member^b^	−1.46	0.44	*0.23*	0.1 –0.55	.001
Social support	−0.08	0.02	*0.92*	0.89–0.96	<.001
Education (years)	0.03	0.06	1.03	0.92–1.15	.605
Type of loss^c^	−0.19	0.48	0.82	0.32–2.11	.685
PTSD vs. resilient (reference)					
Male gender^a^	−0.36	0.42	0.69	0.3–1.59	.390
Assaultive TE	0.15	0.09	1.16	0.97–1.39	.097
Accidental TE	0.21	0.13	1.24	0.96–1.6	.105
Time elapsed since loss (years)	0.04	0.03	1.04	0.99–1.1	.117
Distant family member^b^	0.50	0.43	1.65	0.71–3.82	.244
Social support	−0.05	0.02	*0.95*	0.92–0.99	.005
Education (years)	0.03	0.05	1.03	0.94–1.14	.530
Type of loss^c^	−0.10	0.48	0.91	0.36–2.3	.840
PGD vs. resilient (reference)					
Male gender^a^	−0.42	0.41	0.66	0.29–1.48	.313
Assaultive TE	0.20	0.09	*1.23*	1.02–1.47	.031
Accidental TE	0.12	0.13	1.13	0.88–1.45	.352
Time elapsed since loss (years)	−0.04	0.03	0.96	0.91–1.02	.230
Distant family member^b^	−1.32	0.47	*0.27*	0.11–0.67	.005
Social support	−0.01	0.02	0.99	0.95–1.03	.479
Education (years)	−0.06	0.07	0.95	0.82–1.08	.418
Type of loss^c^	0.33	0.44	1.39	0.59–3.29	.454
PGD vs. PTSD (reference)					
Male gender^a^	−0.05	0.45	0.95	0.39–2.29	.907
Assaultive TE	0.05	0.09	1.05	0.89–1.25	.556
Accidental TE	−0.09	0.13	0.91	0.7–1.18	.487
Time elapsed since loss (years)	−0.08	0.03	*0.92*	0.87–0.98	.005
Distant family member^b^	−1.82	0.49	*0.16*	0.06–0.42	<.001
Social support	0.04	0.02	1.04	0.99–1.08	.089
Education (years)	−0.09	0.07	0.92	0.8–1.05	.196
Type of loss^c^	0.43	0.48	1.53	0.6–3.93	.377

^a^0: female, 1: male. ^b^0: close family member (parent, child, sibling), 1: distant family member (aunt/uncle, grandparent, cousin) or friend. ^c^0: deceased within armed conflict, 1: disappeared within armed conflict

## Discussion

This study sought to identify classes of symptom profiles among Colombians who were exposed to trauma and loss based on symptoms of PGD and PTSD. Four latent classes fitted the data best: a PTSD class characterized by high item probabilities for almost all PTSD symptoms and none of the PGD symptoms, a predominately PGD class marked by high item probabilities for the PGD separation distress symptoms, two other PGD and four PTSD symptoms, a high distress class with high endorsement for nearly all PGD and PTSD symptoms, and finally, a resilient class characterized by moderate to low item probabilities for all symptoms. The four classes were distributed fairly similarly with the high distress class representing the largest class (27.8%) and the predominately PTSD-class representing the smallest (23.3%). These findings replicate previous results of the study by Nickerson and colleagues [19] who also found a four-class solution with very similar symptom profiles as the optimal fit for their data. Findings of both studies therefore indicate that among people exposed to trauma and loss, subgroups are separable both by symptom severity and dominance of a particular syndrome (e.g. some with PTSD, others with predominately PGD). Replicating these previous findings suggests that certain symptom profiles may be consistent across different samples.

The PTSD and PGD classes were clearly separable by high item probabilities for the separation distress symptoms in the PGD-class as opposed to low item probabilities for these symptoms in the PTSD-class, with separation distress being the defining and unique feature of PGD [2]. The identification of a PTSD and a predominately PGD class provides further evidence that PTSD and PGD are distinguishable syndromes that can occur independently of each other. The PTSD and the PGD class differed from each other such that members of the PGD class were more likely to have experienced the loss of a close relative and that less time had elapsed since the loss. This was in line with our hypothesis and supports previous evidence that a close relationship to the person lost is one of the major risk factors for the development of PGD rather than for PTSD [13, 50]. Also, the association of time since loss with membership to the PGD-class indicated that PGD in particular may diminish as time progresses which is in accordance with a recent longitudinal study [51].

Contrary to our initial hypothesis that PTSD would be predicted by a higher exposure to traumatic events, membership to the PTSD class was predicted only by less perception of social support in comparison to the resilient class when controlled for other loss- and trauma-related characteristics. Only a non-significant association (*p* < .10) with exposure to assaultive traumatic events was found. Given the assumed dose-response relationship between traumatic events and PTSD this preliminary finding is rather surprising and should be addressed in a replication study.

Participants in the high distress class had been exposed to the highest amount of trauma, the loss of a close relative and were likely to not have had access to valuable resources such as social support. Rather than being characterized by symptoms specific to a diagnostic category, participants were marked by intense overall emotional distress. This may be indicative of profound impairment in social, occupational, and potentially interpersonal functioning and stresses the necessity to introduce a disorder category that corresponds to the clinical picture of persons with extreme exposure to adverse events. This clinical picture may be captured by Complex PTSD, a disorder marked by disturbances of affect, self-concept and interpersonal functioning in addition to the core PTSD symptoms and is likely to be introduced as novel clinical category within the ICD-11 [4]. Further research on this issue would be necessary.

Despite the atrocities experienced, there was a resilient class comprising about a fifth of the sample. The resilient class showed low item response probabilities for all symptoms. Compared to the high distress class, participants in this class were more likely to be male, perceived more social support, had experienced less assaultive or accidental events and were more likely to have lost a distant family member or friend than a close relative. These findings are in line with previous studies demonstrating that these factors are associated with resilience [52, 53]. Nickerson et al. [19] found their resilient class to comprise nearly half of the sample and concluded that adaption was the normative response to trauma and bereavement. Participants in the current sample were, however, highly exposed to traumatic events as evidenced by an event load twice as high and to an ongoing armed conflict continuing to cause victims and generating fear on a daily basis. Evidence suggests that an ongoing conflict leads to poorer mental health outcomes among trauma survivors [54] which may serve as an explanation as to why the resilient class in this sample was smaller.

The beneficial effects of social support in maintaining mental health regardless of stressful experiences have been documented in a number of studies [26, 27]. In line with these findings, less perceived social support was, relative to the resilient class, associated with membership in the PTSD and high distress class. In both classes, high item probabilities were found for “feeling detached” and “loss of interest”. If these symptoms are pervasive, they may limit the individual’s social contacts and hence the perception of social support from others. It is also possible that symptom severity increases when no social network is available to process stressful experiences. Contrasting previous findings that show an association between lack of social support and PGD symptom severity [51, 53], the amount of perceived social support did not predict membership to the PGD class in this study.

The type of the loss (deceased vs. disappeared) did not have any effect on class membership. This finding may be attributable to the fact that relatives of disappeared persons and bereaved individuals did not differ from each other with regard to their mental health response in this sample [28]. Evidence regarding differences between mental health responses of relatives of disappeared persons and bereaved individuals is, however, still inconclusive and future research should address this issue further.

Unfortunately, we did not assess posttraumatic and grief-related cognitions, which could have provided valuable insights into the role of cognitive appraisals in maladaptive coping with traumatic events and bereavement. A recent study investigating symptom profiles by PGD and depression found that when age, education, and time since loss were controlled for, cognitive variables were the only factors contributing to membership to the symptomatic classes, stressing the importance of cognitions in maintaining elevated distress [55].

### Limitations

Some limitations should be considered when interpreting the results. Data assessment was based on self-report measures rather than clinical interviews allowing for overestimation of symptom severity. The measures were not validated for use in Colombia. However, the instruments have good psychometric properties and have been widely used in different cultural settings. The cross-sectional nature of this study does not allow us to draw conclusions about causal relationships. Furthermore, participants in this study were internally displaced persons affiliated with our partner organization. Findings may therefore not be generalizable to victims of human rights violations in other contexts. Finally, it is possible that the inclusion of other mental disorders that have frequently found to be comorbid with PGD and PTSD (e.g. depression or anxiety) impact the pattern of classes and the allocation of participants to the classes. Nevertheless, the classes identified are in accordance with previous research in other cultural contexts, which strengthens the validity of the results.

## Conclusion

People exposed to trauma and loss are at high risk for the development of mental disorders such as PTSD and PGD. This study shows that subgroups which are separable both by symptom severity and type of emotional response can be identified. The evidence presented suggests an association between class membership and gender, level of exposure to assaultive and other traumatic events, relationship to the person lost (close vs. distant family member/ friend), time elapsed since the loss, and also the amount of social support perceived. These variables represent a range of risk factors that may help identifying subgroups of trauma and loss exposed individuals at high risk for specific symptom patterns. The results also confirm that there is considerable heterogeneity in response to trauma and loss but that certain symptom profiles may be consistent even across different samples.

Extensive research has focused on the treatment of symptoms of PTSD in the aftermath of violent conflicts [56–58]. Considerably less research has engaged in developing and evaluating combined intervention programs for both PTSD and PGD [59, 60]. The high amount of people experiencing PGD and PTSD after exposure to loss and trauma, however, make the current need for interventions addressing both disorders evident. Furthermore, research should investigate the treatment sequencing in comorbid PGD and PTSD (integrated, sequential, parallel or single diagnosis) or whether treatment that helps with PGD may result in an improvement of PTSD (and vice versa). The current findings have valuable implications for identifying distressed subgroups and tailoring interventions to the patient’s symptom profile.

## Abbreviations

A-BIC: Adjusted Bayesian Information Criterion
AIC: Aikaike Information Criterion
BIC: Bayesian Information Criterion
BLRT: Bootstrapped likelihood ratio test
DSM: Diagnostic and Statistical Manual of Mental Disorders
DUKE-UNC: Duke University North Carolina
ICD: International Classification of Diseases
LCA: Latent Class Analysis
LRT: Likelihood Ratio Test
NGO: Non-Governmental Organisation
OR: Odds Ratio
PCL-C: PTSD Checklist-Civilian version
PG-13: Prolonged Grief Disorder-Scale
PGD: Prolonged grief disorder
PTSD: Posttraumatic stress disorder
SE: Standard Error
VLMR-LRT: Vuong-Lo-Mendell-Rubin Likelihood Ratio Test
